# Dietary Fucose Affects Macrophage Polarization and Reproductive Performance in Mice

**DOI:** 10.3390/nu13030855

**Published:** 2021-03-05

**Authors:** Ekaterina A. Litvinova, Victoria D. Bets, Natalya A. Feofanova, Olga V. Gvozdeva, Kseniya M. Achasova, Elizaveta L. Alperina, Elena N. Kozhevnikova

**Affiliations:** 1 Scientific-Research Institute of Neurosciences and Medicine, 630117 Novosibirsk, Russia; iph@physiol.ru (E.L.A.); e.l.alperina@physiol.ru (E.L.A.); 2 Siberian Federal Scientific Centre of Agro-BioTechnologies of the Russian Academy of Sciences, Krasnoobsk, 630501 Novosibirsk Region, Russia; 3 Faculty of Veterinary Medicine, Novosibirsk State Agrarian University, 630039 Novosibirsk, Russia; 4 Research Institute of Fundamental and Clinical Immunology, 630099 Novosibirsk, Russia; 5 Institute of Molecular and Cellular Biology, The Siberian Branch of the Russian Academy of Sciences, 630090 Novosibirsk, Russia

**Keywords:** Mucin 2, embryo implantation, fucose, macrophages, immune response, pregnancy

## Abstract

Intestinal mucus protects epithelial and immune cells from the gut resident microorganisms, and provides growth-promoting factors as mucus-derived O-glycans for beneficial bacteria. A lack of intestinal protective mucus results in changes in the commensal microflora composition, mucosal immune system reprogramming, and inflammation. Previous work has shown that fucose, the terminal glycan chain component of the intestinal glycoprotein Mucin2, and fucoidan polysaccharides have an anti-inflammatory effect in some mouse models of colitis. This study evaluates the effect of fucose on reproductive performance in heterozygous mutant *Muc2* female mice. We found that even though *Muc2^+/−^* females are physiologically indistinguishable from C57Bl/6 mice, they have a significantly reduced reproductive performance upon dietary fucose supplementation. Metagenomic analysis reveals that the otherwise healthy wild-type siblings of *Muc2^−/−^* animals have reduced numbers of some of the intestinal commensal bacterial species, compared to C57BL/6 mice. We propose that the changes in beneficial microflora affect the immune status in *Muc2^+/−^* mice, which causes implantation impairment. In accordance with this hypothesis, we find that macrophage polarization during pregnancy is impaired in *Muc2^+/−^* females upon addition of fucose. Metabolic profiling of peritoneal macrophages from *Muc2^+/−^* females reveals their predisposition towards anaerobic glycolysis in favor of oxidative phosphorylation, compared to C57BL/6-derived cells. In vitro experiments on phagocytosis activity and mitochondrial respiration suggest that fucose affects oxidative phosphorylation in a genotype-specific manner, which might interfere with implantation depending on the initial status of macrophages. This hypothesis is further confirmed in BALB/c female mice, where fucose caused pregnancy loss and opposed implantation-associated M2 macrophage polarization. Taken together, these data suggest that intestinal microflora affects host immunity and pregnancy outcome. At the same time, dietary fucose might act as a differential regulator of macrophage polarization during implantation, depending on the immune status of the host.

## 1. Introduction

The gastrointestinal (GI) microbiota is extensively involved in a variety of physiological processes, including the regulation of host metabolism, and immune function [1,2,3,4,5,6,7,8]. Its disruptions are linked to such disorders as obesity, atherosclerosis, diabetes, inflammatory bowel disease (IBD), colorectal cancer (CRC), and neurobehavioral abnormalities [4,7,8,9]. The gut microbiota plays an important role during pregnancy, which is associated with significant alterations in the host metabolism and immune system. Increasing evidence suggests that changes in the structure and function of the maternal intestinal microbiota may significantly affect pregnancy progression [7,10,11,12].

The inner intestinal surface is protected by the gel-like mucus produced by the specialized goblet cells and forms a physical barrier between the microorganisms and epithelial and immune cells [13,14]. Mucus is composed of heavily glycosylated proteins called mucins, among which Mucin 2 (MUC2) is the main secretory mucin in the small and large intestine [8,15,16,17,18]. The structural diversity and monosaccharide composition of MUC2 O-glycans have significant effects on mucus barrier function, the gut microbiome, and the immune status [8,15,16,17,19,20,21]. *Mucin 2 (Muc2*) gene knockout in mice results in immune cell reprogramming, carcinogenesis, and alterations in the gut microbiota [18,22,23,24]. Mice with the *Muc2* deficiency showed clinical and morphological signs of colitis [18,24,25]. They developed intestinal and rectal tumors more frequently than the wild-type controls [18,22,24]. Increased susceptibility to experimental colitis and carcinomas have been also observed in mice lacking core 1- and core 3-derived O-glycans [26,27].

Generally, *Muc2* knockout mice suffer from diarrhea, bloody stool, low weight, and tend to develop rectal prolapses by the age of 2–3 months. These factors have a strong negative impact on the course of pregnancy, so that most of the homozygous mutant females do not produce progeny [28,29]. Apparently, the physical condition of *Muc2* knockout females prevents normal reproduction. However, the impact of microbiome, metabolism, and immune system alterations in pregnancy development cannot be excluded. It has been shown that the immune system was generally activated upon *Muc2* knockout, which might change the properties of the distinct immune cell types involved in pregnancy progression [30,31,32]. For instance, colitis is associated with altered gene expression profiles in macrophages that are involved in the maintenance of mucosal immune system homeostasis and prevent damaging inflammation [33,34,35,36]. Likewise, beneficial microbes are potential modulators of macrophage functions as a number of authors demonstrates that probiotics like *Lactobacilli* or microflora-derived metabolites like butyrate reduce inflammation and restore barrier function [28,37,38,39,40]. Thus, changes in metabolic profile and inflammatory signals associated with impaired microflora are key to macrophage polarization [41,42,43,44]. At the same time, macrophages assist pregnancy and undergo complex reprogramming during implantation [45,46,47]. M1 macrophages are found during early embryo development and embryo attachment, where M1/M2 transition occurs. M2 macrophages support placenta development and pregnancy maintenance. Macrophages re-polarize to M1 by the end of pregnancy and support parturition [47,48]. Therefore, intestinal inflammation and microflora imbalance during pregnancy might affect implantation and embryo development.

The major bacteria growth-promoting factors in the intestinal mucus-derived O-glycans are often reduced upon colitis [21,49,50,51]. It is proposed that dietary glycans, like fucoidan, can partially substitute for the lack of the natural O-glycoside chains and have an ameliorating effect on inflammation and microflora composition [7,52,53,54,55]. However, to date, the impact of the external supplementation with mucus-derived glycans and their terminal monosaccharide fucose on pregnancy development remains unclear. Therefore, the aim of the present study was to evaluate the effect of fucose on pregnancy and reproductive performance in C57Bl/6, BALB/c, and *Muc2^+/−^* mice that differ in immune status.

## 2. Materials and Methods

### 2.1. Animal Housing

The study was conducted in the Center for Genetic Resources of Laboratory Animals at the Federal Research Center Institute of Cytology and Genetics of The Siberian Branch of the Russian Academy of Sciences (ICG SB RAS), a unique identifier of the project RFMEFI62117 × 0015. All procedures were conducted under Russian legislation according to Good Laboratory Practice standards (directive # 267 from 19 June 2003 of the Ministry of Health of the Russian Federation), inter-institutional bioethical committee guidelines, and the European Convention for the protection of vertebrate animals used for experimental and other scientific purposes; all procedures were approved by the bioethical committee, protocol #18.6 (14 October 2013). All animals used had specific pathogen free (SPF) status, which was tested quarterly according to FELASA recommendations [56].

The experiments were performed in adult male and female mice of the C57BL/6JNskrc (C57BL/6), *Muc2^+/−^*, and *Muc2^−/−^* strains. *Muc2^+/−^* mice were generated by crossing *Muc2^tm1Avel^/Muc2^tm1Avel^* (*Muc2^−/−^* on C57BL/6 genetic background) males to C57BL/6 females. *Muc2^−/−^* mice were obtained from the Federal Research Centre “Fundamentals of Biotechnology” of the Russian Academy of Sciences (Moscow, Russia).

All animals were weaned at three weeks of age and kept in groups of same-sex siblings in open cages until 10–12 weeks. They were then placed in individually ventilated cages (outside cage dimensions: 13.5” (343 mm) L × 11.5” (292 mm) W (front) × 6.1” (155 mm) H; cage floor area: 75 square inches (484 square centimeters), Optimice (AnimalCare Systems, Centennial, USA. All animals were housed under a 14 h/10 h light/dark photoperiod (light off at 16.00) with 22–24 °C temperature, 30–60% humidity, and 10 air changes per hour; food (ssniff Spezialdiaeten GmbH, Soest, Germany) and water were provided ad libitum. Individually ventilated cages were supplied with birch sawdust as litter and plastic cups as shelter. Cages were replaced every 7 days, except for during the first 24 h after delivery. All samples were collected between 12:00 and 16:00 (light time period).

### 2.2. Experimental Groups

First mating. Female mice of each genotype (C57BL/6, *n* = 18, and *Muc2^+/−^*, *n* = 18) were placed in cages (*n* = 3 of the same genotype per cage, 12 cages in total). A mature male mouse (12–14 weeks old) was introduced into each cage with female mice for 4 days. C57BL/6 males were introduced to the C57BL/6 females, homozygous *Muc2* mutant (*Muc2^−/−^*) males were introduced to *Muc2^+/−^* females. After a male mouse was removed, each female was placed in an individual cage and randomly assigned to receive either regular drinking water (“Control” groups of C57BL/6 or *Muc2^+/−^*, *n* = 9/group), water with 0.05% fucose (Biosynth Carbosynth, Compton, UK) (“Fucose” groups of C57BL/6 or *Muc2^+/−^*, *n* = 9/group). The supplementation with fucose continued throughout.

Second mating. Two weeks after the pups weaning, the same *Muc2^+/−^* female mice were placed in 12 cages (*n* = 3 per cage) with respect to the groups they were assigned in the first mating. A mature *Muc2* heterozygous male mouse (*Muc2^+/−^*, 12–14 weeks old) was introduced into each cage with female mice for 2 days. After a male mouse was removed, the females were placed in individual cages and received water or monosaccharide solutions in the same concentration, so that each group received the same monosaccharide as it did in the first mating. The females were sacrificed and used for sample collection and reproductive system examination on the 12th day after removing a male mouse, gestation day (GD) 12–14.

Co-housing experiment. Three 5–6 weeks-old C57BL/6 female mice of each genotype were placed in the same cage with three 5–6 weeks-old *Muc2^+/-^* female mice (*n* = 18 in total for each genotype, making 6 cages in total) and-co-housed for 4 weeks. After that, females were separated according to their genotype and mated to male mice of the same genotype for 3 days. Then all female mice were placed back in the same cage in the same order (3 C57BL/6 and 3 *Muc2^+/−^* females), and co-housed until 17–19 d.p.c. After that, pregnant females were housed individually. On the day the male mice were removed, 18 female mice (3 cages) received 0.05% Fucose in drinking water until labor.

BALB/c mating. Female mice of each genotype (C57BL/6, *n* = 18, and BALB/c, *n* = 18) were placed in cages (*n* = 3 of the same genotype per cage, 12 cages in total). A mature male mouse (12–14 weeks old) was introduced into each cage with female mice for mating. C57BL/6 males were introduced to the C57BL/6 females, BALB/c males were introduced to BALB/c females. Females with vaginal plugs were placed in an individual cage and randomly assigned to receive either regular drinking water (“Control” groups of C57BL/6 or BALB/c, *n* = 9/group) or water with 0.05% fucose (Biosynth Carbosynth, UK) (“Fucose” groups of C57BL/6 or BALB/c, *n* = 9/group). The supplementation with fucose continued throughout pregnancy. Reproductive performance data for C57BL/6 mice was combined from the first mating and BALB/c mating. 

### 2.3. Sample and Tissue Collection

Blood samples from the *Muc2^+/−^* dams were collected by the orbital sinus puncture without using anesthesia as it can affect the immune cell count, due to glucocorticoid reaction [57,58]. For blood collection, the eyes of the test mice were treated with a drop of an ophthalmic anesthetic (0.5% proparacaine hydrochloride ophthalmic solution, Alcon Laboratories, Camberley, UK). After blood collection, the mice were euthanized using CO_2_ inhalation. Immediately after, reproductive systems of *Muc2^+/−^* dams were examined, embryos and yellow bodies were counted. Intestinal contents were collected from *Muc2^+/−^* dams in a sterile 1.5-mL plastic tube, snap-frozen in liquid nitrogen, and stored at −70 °C until further analysis. For the macrophage isolation, separate matings were performed, the dams (*N* = 6–12) were sacrificed at 5GD, and peritoneal macrophages were isolated. For this, females were euthanized by decapitation, and the peritoneal cavity was washed with 5 mL of sterile ice-cold PBS. Suspension of peritoneal cells was collected by a syringe and centrifuged for 5 min at 1500 rpm. Cell pellets were resuspended in DMEM medium with 10% FBS and left to adhere in 24-well culture plate (Corning) at 1 × 10^6^ cells per well for 1 h in CO_2_ incubator at 37 °C. Non-adherent cells were removed by washing twice with PBS medium. Adherent macrophages were used for further assays. To test the effect of fucose on cellular metabolism and phagocytosis, each sample of peritoneal macrophages was divided into two wells and incubated with or without 0.1% L-fucose for 12 h. After that, cells were washed with medium without fucose and used for downstream applications.

### 2.4. Metagenomic Analysis

DNA was purified from intestinal contents using QIAamp DNA Stool Mini Kit (Qiagen, Hilden, Germany) according to the manufacturer’s recommendations (N(C57BL/6) = 4, N(*Muc2^+/+^*) = 4). The *16S rRNA* 16SV3-V4 region was amplified using barcoded primers. All PCR reactions were carried out using Phusion^®^ High-Fidelity PCR Master Mix (New England Biolabs, Ipswich, USA). PCR products was mixed in equal ratios. The mixture of PCR products were purified with Qiagen Gel Extraction Kit (Qiagen, Hilden, Germany). The libraries were generated with NEBNext^®^ UltraTM DNA Library Prep Kit for Illumina and analyzed by the Illumina platform. Sequences analysis were performed by Uparse software. Sequences with ≥97% similarity were assigned to the same OTUs. Beta diversity analysis was used to evaluate differences between samples in species complexity. Beta diversity of both: Weighted and unweighted unifrac were calculated by QIIME software (Version 1.7.0). Cluster analysis was preceded by principal component analysis (PCA), which was applied to reduce the dimension of the original variables using the FactoMineR package and ggplot2 package in R software (Version 2.15.3). Normalized reads for each OUT were used to show the average top ten taxa in each group and calculate differences in taxa abundance between groups. 

### 2.5. Flow Cytometry Analysis (FC)

Red blood cells were lysed with ammonium chloride buffer (0.15 M NH_4_Cl; 0.01 M NaHCO_3_; 0.001 M EDTA) for 10 min at room temperature, and leukocytes were centrifuged at 1500 rpm +4 °C for 5 min. White blood cells were washed twice with 2% BSA in phosphate-buffered saline (PBS), resuspended in staining buffer (1% BSA, 0.1% sodium azide in PBS), and diluted up to the concentration of 1000–1200 cells/μL. Then, 250 μL of cell suspension were stained with PE-Cy7-CD45 (Rat IgG2b, k, clone 30-F11), FITC-CD19 (Rat IgG2a κ, clone 6D5), PE-anti-CD3ε (Hamster, clone 145-2C11) and PE-CD3ε (Hamster, clone 145-2C11), FITC-CD4 (Rat IgG2b κ, clone GK1.5), PE/Cy7-CD8a (Rat IgG2a κ, clone 53–6.7) anti-mouse antibodies (all BioLegend, San Diego, USA) for 120 min at 4 °C in the dark and then samples were analyzed using Guava easyCyte Flow Cytometer (Merck KGaA, Darmstadt, Germany). For analysis, 25,000 lymphocytes were counted in each sample. The number of blood CD4^+^-and CD8^+^-cells was expressed as a ratio of CD4^+^- and CD8^+^- percentages of CD3^+^-cells. To determine leukocyte number, whole blood samples were stained with Türk’s solution (Merck, USA), leukocytes were counted in a counting-chamber and expressed as 10^6 per ml of blood.

250 μL of macrophage cell suspension were stained with FITC-F4/80 (Rat IgG2b, k, clone EMR1), PE-CD209a (Mouse IgG2c, clone MMD3), APC-anti-CD86 (Rat IgG2a, clone GL-1) (all BioLegend, USA) for 60 min at 4 °C in the dark and then samples were analyzed using BD FACSCanto II Flow Cytometer. During analysis, we first isolated single cells (singlets), which were used to isolate macrophages positive for F4/80 marker. This cell population was further used to identify M1 (positive for CD86) and M2 macrophages (positive for CD209).

### 2.6. Macrophage Phagocytosis Assay

For in vitro phagocytosis, C57BL/6 and *Muc2^+/−^* dams were used (*n* = 5 for each genotype). Each macrophage preparation was divided into two samples and either incubated or not with 0.1% fucose for 12 h. Two μL of fluorescent particle suspension (yellow-green latex beads 2-μm in size, Sigma-Aldrich, St. Louis, USA) were added per each well of 24-well culture plate with adherent macrophages in final concentration 2 μL/ml media. Plates were placed or in CO_2_ incubator at 37 °C for 1 h. After that, media was removed, cells were washed 3 times with PBS, detached from the plate with a plastic scraper, resuspended in PBS, and used for flow cytometry analysis. 

### 2.7. Mitochondrial Respiration and Anaerobic Glycolysis

Oxygen consumption rate (OCR) and extracellular acidification rate (ECAR) were measured using Seahorse XF Cell Mito Stress Test (Agilent, Santa Clara, CA, US). C57BL/6 (*n* = 5) and *Muc2^+/−^* (*n* = 6) dams were used for macrophage isolation. Each cell sample was divided into two and either incubated or not with 0.1% fucose for 12h. After that, cells were seeded onto an XFe24 bioflux plate (Seahorse Bioscience, North Billerica, USA) at a pre-optimized final concentration of 5 × 10^5^ cells/well. Mitochondrial function was measured as OCR after injections of 0.5 μM oligomycin (ATP synthase inhibitor), 1 μM FCCP (electron transport chain accelerator), and 1 μM anti-mycin A (complex III inhibitor) plus 1 μM rotenone (complex I inhibitor), according to the manufacturer’s instructions. ECAR was measured simultaneously in the same experiment. Seahorse XFe Wave Software (Seahorse Bioscience, North Billerica, USA) was applied to analyze the data.

### 2.8. Data Analysis and Statistics

The data were tested for normality using the Kolmogorov-Smirnov test. Normally distributed data were processed using analysis of variance (ANOVA) and Student’s *t*-test test. Not normally distributed data were processed using Mann–Whitney *U* test. Ln-transformation was applied to macrophage percentages and SeaHorse analysis data, followed by the Kolmogorov-Smirnov test prior to ANOVA. Reproductive performance, pre-implantation losses, and post-implantation losses were analyzed using Fisher exact test. In vitro phagocytosis and mitochondrial respiration were analyzed using paired Student’s *t*-test.

## 3. Results

### 3.1. The Effect of Fucose on Reproductive Performance of Muc2^+/−^ and C57BL/6 Females

**Figure 1:**
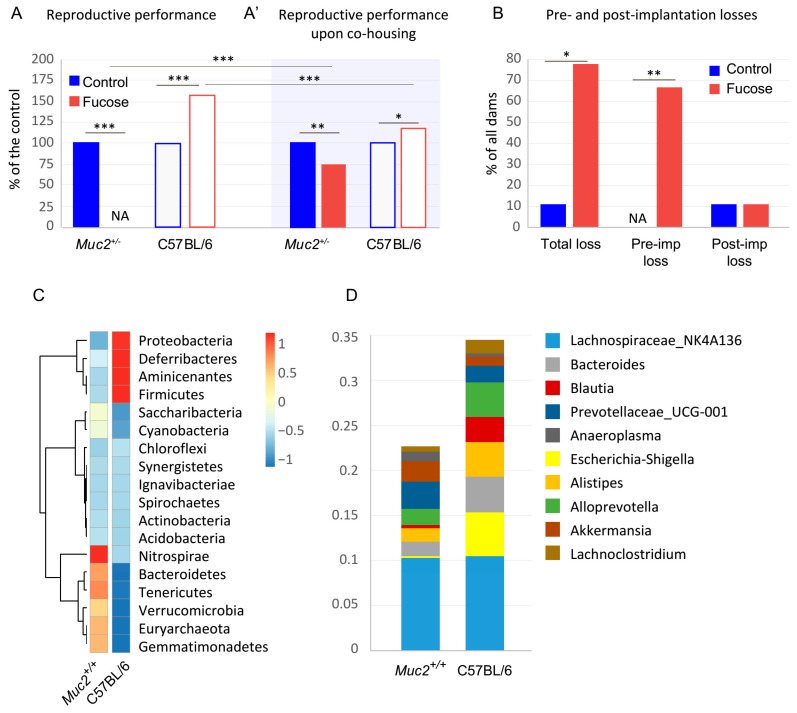
Fucose affects reproductive performance in *Muc2^+/^*^−^ and C57BL/6 dams. (**A**). Reproductive performance in ♀*Muc2^+/^*^−^ × ♂*Muc2*^−*/*−^ and in C57BL/6 matings. *** *p* < 0.001, Fisher exact test; N(*Muc2^+/^*^−^ dams) = 9, N(C57BL/6 dams) = 18. (**A’**). Reproductive performance of *Muc^+/^*^−^ and C57BL/6 female mice upon co-housing. * *p* < 0.05, ** *p* < 0.01, *** *p* < 0.001, Fisher exact test; N(*Muc2^+/^*^−^ dams) = 9, N(C57BL/6 dams) = 9 in each group. (**B**). Pre- and postimplantation losses in in ♀*Muc2^+/^*^−^ x ♂*Muc2^+/^*^−^ matings. “Fucose” vs. “Control”: * *p* < 0.05, ** *p* < 0.01, *** *p* < 0.001, Fisher exact test; N(dams) = 9 in each group. (**C**). Cluster analysis of OTU in *Muc2^+/+^* (N = 4) and C57BL/6 (N = 4) animals. (**D**). Percent of top 10 OTU present in the intestinal content.

As *Muc2* homozygous mutant females do not produce progeny, we aimed to overcome this issue by adding fucose to the drinking water, but revealed no positive effect (data not shown). However, we noticed that *Muc2^+/^*^−^ females routinely used to maintain mutant mouse colony did not produce progeny upon the same treatment. Thus, we first evaluated the effect of monosaccharide fucose on the reproductive performance of the *Muc2^+/^*^−^ females. We found that fucose decreased reproductive performance of test females crossed to *Muc2^-/-^* males (first mating). The addition of fucose resulted in no offspring (23 vs. 0, Fisher exact test *p* < 0.001, Figure 1A). 

We further tested whether this effect was exclusive to the females of *Muc2^+/−^* genotype. Therefore, we supplemented the drinking water of C57BL/6 females with fucose, along with the control group that received drinking water. On the contrary, the reproductive performance of the C57BL/6 females was significantly higher upon fucose treatment as compared to the untreated control (Fisher exact test, *p* < 0.001, Figure 1A). This data demonstrates that the effect of fucose was opposite in the two groups and depended on the genotype.

We then questioned which state of pregnancy was affected by the fucose. In order to exclude the effect of germ-line deficiency in *Muc2^−/−^* males, we used *Muc2^+/−^* males in the second mating with the *Muc2^+/−^* females. We scarified dams (N = 9) in the second mating on the 12–14 d.p.c. to count embryos and yellow bodies. We found that fucose elevated pre-implantation losses: All females in the “Fucose” group had yellow bodies, but only 3 out of 9 females had any embryos upon fucose treatment in comparison to the control group, where all of the nine females with yellow bodies had at least one embryo (Fisher exact test, *p* < 0.01, Figure 1B). Post-implantation losses were insignificant in the “Fucose” group, but total losses were higher in this group as compared to the control (Fisher exact test, *p* < 0.05, Figure 1B). 

It was shown previously that *Muc2^+/−^* animals are physiologically healthy and cannot be distinguished from C57BL/6 based on intestinal morphology and inflammatory parameters [59]. However, these animals were co-housed with their mutant littermates and might have microflora shifts associated with *Muc2* mutation as it was shown that co-housing can affect microbiome [60]. Thus, we performed a metagenomic analysis of the intestinal microbiome of wild-type littermates co-housed with *Muc2* mutant mice and C57BL/6 animals housed separately in individually ventilated cages. We found that, indeed, co-housing with *Muc2* mutant mice affected the microbiome of their wild-type littermates compared to C57BL/6 (Figure 1C). For instance, *Blautia* and *Escherichia* species were significantly reduced upon co-housing with *Muc2* mutants as revealed by a Mann–Whitney *U* test (Z = 2.16, *p* < 0.05 for both). Thus, the deficiency in beneficial microbes might contribute to the effect of fucose on pregnancy in *Muc2^+/−^* dams.

To confirm the effect of microflora on reproduction in *Muc2^+/−^* animals, we co-housed *Muc2^+/-^* and C57BL/6 female mice before and during pregnancy. We found that co-housing with C57BL/6 mice significantly increased reproductive performance in *Muc2^+/−^* dams after fucose treatment (Fisher exact test, *p* < 0.001, Figure 1A,A′); however, it did not completely restore it (Fisher exact test, *p* < 0.01, Figure 1A’). At the same time, co-housing C57BL/6 dams with *Muc2^+/−^* mice attenuated the beneficiary effect of fucose on pregnancy (Fisher exact test, *p* < 0.001, Figure 1A,A′), though it was not fully eliminated (Fisher exact test, *p* < 0.05, Figure 1A’). Together this data shows that equilibration of microflora between *Muc2^+/−^* and C57BL/6 dams by co-housing mitigates the differences in their reproductive performance. It confirms our hypothesis that the reported above reproductive failure of *Muc2^+/−^* dams upon fucose treatment originates from the microbiome composition of these otherwise healthy animals.

### 3.2. The Effect of Fucose on the Systemic Immune Response

**Figure 2:**
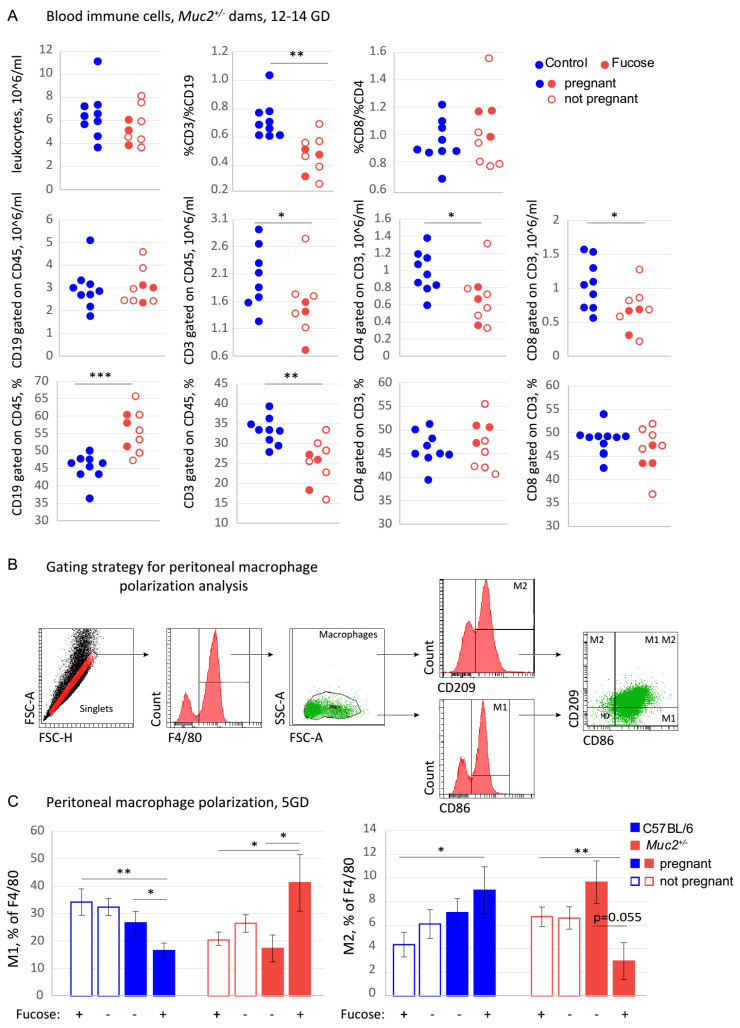
Blood immune cells ratios in *Muc2^+/^^−^* dams and peritoneal macrophage polarization in C57BL/6 and *Muc2^+/^^−^* dams. (**A**). Blood immune cells: Absolute numbers, percentages, and percentage ratios. “Fucose” vs. “Control”: * *p* < 0.05, ** *p* < 0.01, *** *p* < 0.001; Student’s *t*-test, N(dams) = 9 in all groups. (**B**). Gating strategy for peritoneal macrophage polarization analysis. (**C**). Percentage of M1 and M2 macrophages with and without fucose treatment in C57BL/6 and *Muc2^+/^^−^* dams.: * *p* < 0.05, ** *p* < 0.01, N = 6–10 in each group, Student’s *t*-test.

An appropriate and balanced immune reaction is important for successful implantation and embryo development during pregnancy [61]. As intestinal bacteria do not directly affect embryo development and implantation, we questioned whether microflora might affect pregnancy via modulation of the immune system. To test this hypothesis, we used FC analysis that quantifies the major lymphocyte populations in the blood: T-cells (CD45^+^CD3^+^) and B-cells (CD45^+^CD19^+^) and subpopulations of T-cells: T-helpers (CD3^+^CD4^+^) and T-killers (CD3^+^CD8^+^). FC did not reveal any effects of the monosaccharide on the total count of leukocytes, the absolute numbers of CD45^+^CD3^+^ and CD3^+^CD4^+^, the percentages of CD3^+^CD4^+^and CD3^+^CD8^+^, and the percentage ratio of CD3^+^CD4^+^ to CD3^+^CD8^+^ lymphocytes (Figure 2A). However, there was a strong effect of fucose on the percentage ratio of T-cells to B-cells. Fucose significantly reduced the CD3/CD19 percentage ratio as compared to the “Control” (Student’s *t*-test: “Fucose”: *p* = 0.001). Fucose also significantly reduced the absolute numbers of CD45^+^CD3^+^, CD3^+^CD8^+^, and CD3^+^CD4^+^ (Student’s *t*-test: “Fucose”: *p* < 0.05 for each cell type) lymphocytes, and the percentage of CD45^+^CD3^+^ lymphocytes (Student’s *t*-test: “Fucose”: *p* < 0.01, Figure 2A). At the same time, fucose treatment resulted in a significant increase of CD45^+^CD19^+^ lymphocytes percentage (Student’s *t*-test: “Fucose”: *p* < 0.001).

These data demonstrate that fucose affects major blood lymphocyte populations. Therefore, it might also influence other immune cell types involved in implantation and pregnancy. Since macrophages are well known to provide regulatory signals during implantation, we tested the effect of fucose on the polarization of peritoneal macrophages from *Muc2^+/−^* and C57BL/6 female mice. Pregnant and non-pregnant dams of both genotypes at 5GD with and without fucose treatment were used (*n* = 6–10 in each group, eight groups in total). There was a significant interaction of “Genotype”, “Pregnancy” and “Fucose” factors for M1 (F (1, 59) = 12.19, *p* < 0.001) and M2 (F (1, 59) = 9.11, *p* < 0.001) macrophage percentages. After fucose treatment, the percentage of M1 macrophages in C57BL/6 pregnant dams was significantly lower as compared to non-pregnant females (Student’s *t*-test: *p* < 0.01, Figure 2C ). In pregnant C57BL/6 dams, the percentage of M1 macrophages were lower after fucose treatment (Student’s *t*-test: *p* < 0.05, Figure 2C). On the contrary, after fucose treatment, the percentage of M1 macrophages in *Muc2^+/−^* pregnant dams was significantly higher as compared to non-pregnant females (Student’s *t*-test: *p* < 0.05, Figure 2C). In pregnant *Muc2^+/−^* dams, the percentage of M1 macrophages were higher after fucose treatment (Student’s *t*-test: *p* < 0.05, Figure 2C). Fucose also exerted an opposing effect on M2 macrophages in the two genotypes. After fucose, the percentage of M2 macrophages increased upon pregnancy in C57BL/6 dams (Student’s *t*-test: *p* < 0.05, Figure 2C), but decreased in *Muc2^+/−^* dams (Student’s *t*-test: *p* < 0.05, Figure 2C). In pregnant *Muc2^+/−^* dams, there was a tendency for M2 decrease after fucose treatment (Student’s *t*-test: *p* = 0.055, Figure 2C). These data indicate that fucose affects T- to B-cell ratio and opposes M2 polarization in *Muc2^+/−^* dams, which might contribute to the local immune reactions in the uterus and placenta.

### 3.3. Fucose Regulates Macrophage Activity and Metabolism In Vitro

**Figure 3:**
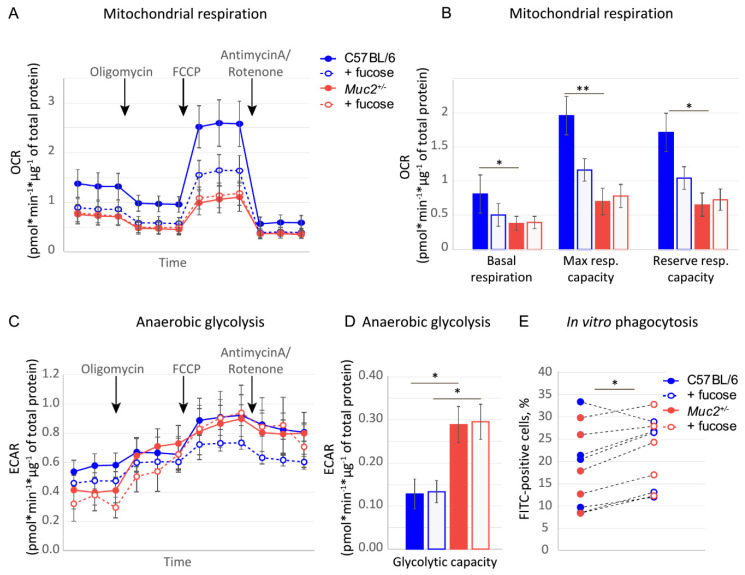
Energy metabolism and phagocytic activity in isolated peritoneal macrophages. (**A**). OCR was measured at basal level followed by the sequential treatment with oligomycin, FCCP, and a mixture of antimycin A and rotenone. Each data point represents average OCR per group per measurement. (**B**). Basal respiration, maximal, and reserve respiratory capacity. “C57BL/s” vs. “*Muc2^+/−^*”: * *p* < 0.05, ** *p* < 0.01, *n* = 5–6, Student’s *t*-test. (**C**). ECAR was measured at basal level followed by the sequential treatment with oligomycin, FCCP, and a mixture of antimycin A and rotenone. Each data point represents average ECAR per group per measurement. (**D**). Glycolytic capacity. “C57BL/s” vs. “*Muc2^+/−^“*: * *p* < 0.05, *n* = 5–6, Student’s *t*-test. (**E**). In vitro phagocytosis of peritoneal macrophages, percentage of cells that captured fluorescent particles. “Fucose” vs. “Control”: * *p* < 0.05, N = 11 in each group, paired Student’s *t*-test.

The effect of fucose on reproduction and macrophage polarization might be either direct or indirect, for instance, via modulation of the microbiome. We used in vitro experiments with primary peritoneal macrophages to understand whether fucose can directly regulate their properties. Metabolic changes accompany macrophage polarization, and might predispose their fate. Thus, we investigated oxidative phosphorylation and anaerobic glycolysis—metabolic signatures of M2/M1 polarization in macrophages isolated from C57BL/6 (*n* = 5) and *Muc2^+/−^* (*n* = 6) dams upon addition of fucose in vitro. We found that fucose generally reduced OCR in C57BL/6-derived macrophages, but this effect was not statistically significant (Figure 3A). At the same time, ANOVA revealed a significant effect of genotype on basal respiration (F(1,18) = 5.7 *p* < 0.05), maximal respiratory capacity (F(1,18) = 3,3 *p* < 0.01), reserved respiratory capacity (F(1,18) = 2.5 *p* < 0.01) and glycolytic capacity (F(1,18) = 18.8, *p* = 0.001). Oxidative phosphorylation was more prominent in C57BL/6-derived macrophages as basal respiration, maximal, and reserved respiratory capacity were higher in this group (Student’s *t*-test: *p* < 0.05, *p* < 0.01, and *p* = 0.01, respectively, Figure 3B). On the contrary, glycolytic capacity was significantly higher in *Muc2^+/−^*-derived cells (Student’s *t*-test: *p* < 0.05, Figure 3C,D). This experiment shows that the metabolic states of macrophages are different in the two genotypes. However, fucose is unable to change these parameters in vitro. We also performed in vitro phagocytosis as a functional test for macrophages from C57BL/6 (*n* = 5) and *Muc2^+/−^* (*n* = 5) dams. Twelve hour-long incubation with fucose significantly increased their phagocytosis activity (paired Student’s *t*-test, *p* < 0,05 for each, Figure 3E), but did not have any genotype-specific effect. Thus, at least in part, fucose might directly regulate macrophage function.

### 3.4. Fucose Affects Reproductive Performance and Macrophage Polarization in BALB/c Mice

**Figure 4:**
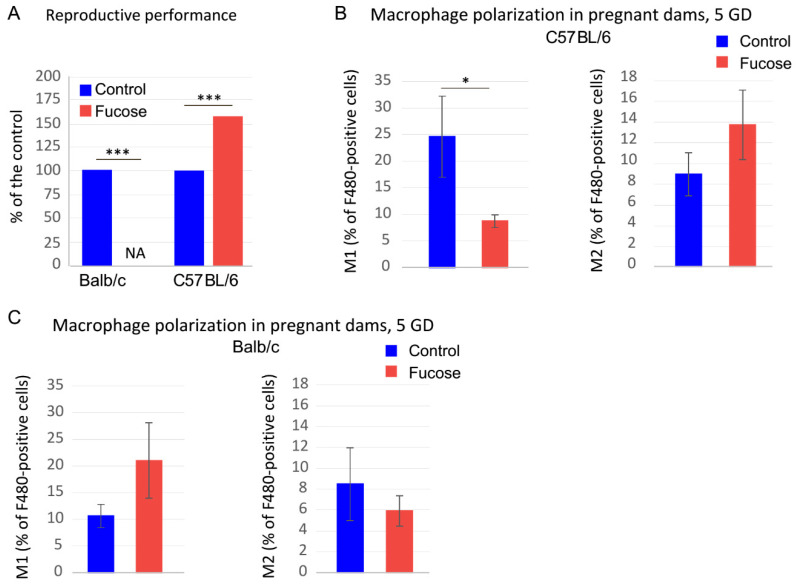
Fucose affects reproductive performance and macrophage polarization in C57BL/6 and BALB/c dams. (**A**). Reproductive performance in C57BL/6 and BALB/c dams upon fucose treatment. *** *p* < 0.001, Fisher exact test; N(dams) = 9 in each group. (**B**). Percentage of M1 and M2 macrophages upon fucose treatment in pregnant C57BL/6 dams. “Fucose” vs. “Control”: * *p* < 0.05, N = 3–4, Mann–Whitney *U* test. (**C**). Percentage of M1 and M2 macrophages upon fucose treatment in pregnant BALB/c dams, N = 3–8.

Since fucose affects macrophages directly, this can cause implantation deficiency in *Muc2^+/−^* dams. In order to test the opposite effect of fucose on macrophages depending on their initial propensity for polarization, we used two prototypical mouse strains with Th1- and Th2-type of the innate immune response: C57BL/6 and BALB/c, respectively. Our experiments revealed that, indeed, fucose strongly reduced reproductive performance in BALB/c dams (Fisher exact test, *p* < 0.001, Figure 4A) so that we received no live progeny upon fucose treatment. At the same time, C57BL/6 dams demonstrated even higher reproductive performance upon fucose treatment (Fisher exact test, *p* < 0.001, Figure 4A). Accordingly, fucose significantly reduced the percentage of M1 peritoneal macrophages at 5GD in pregnant dams (Mann–Whitney *U* test: Z = 2.14, *p* < 0.05, Figure 4B), whereas no such change was observed in BALB/c dams (Figure 4B). In fact, there was a tendency to M1 upregulation upon fucose treatment in BALB/c pregnant dams. Thus, fucose might directly affect macrophage polarization in a predisposed host and affect implantation and pregnancy outcome.

## 4. Discussion

Even though fucosylated glycans play a pivotal role in pregnancy [62], not much is known about the effect of free fucose on embryo development and implantation. Given the fact that fucose affected pregnancy and reproductive performance in *Muc2^+/−^* only, we propose that this effect is not common in wild-type mice, but is associated with *Muc2* mutation. *Muc2* gene encodes for a major intestinal glycoprotein Mucin 2, which forms a gel-like barrier on the apical surface of the intestinal epithelial cells and protects the mucosal immune system from unwanted contact with the intestinal bacteria [22]. Homozygous mutation in this gene results in an inflammatory response, spontaneous colitis, loss of the intestinal barrier integrity, and development of adenocarcenomas [22,23]. Moreover, mucin depletion and inflammatory reaction cause substantial changes in the intestinal microflora in *Muc2* mutant mice [24]. At the same time, *Muc2^+/−^* animals appear normal both in terms of general physiology and in the morphology and function of the intestine [59]. However, co-housing of mice with different microbiomes are known to affect intestinal bacterial communities [60], therefore *Muc2* mutant mice’ microflora partially equilibrated with their otherwise physiologically normal siblings, as was shown by metagenomic analysis. Some crucial beneficial bacteria like *Escherichia* and *Blautia* were significantly downregulated upon co-housing with the mutant mice. For instance, *Blautia* is one of the major butyrate producers in the intestine, and its reduction is associated with Crohn’s disease [63,64]. Butyrate, in turn, is known to promote M2 macrophage polarization [65] and is associated with the decrease of pregnancy complications in rat models [66]. Thus, macrophage propensity to polarize might originate from the background immune-system-microflora interactions via the changes in immunity or metabolism. Our in vitro experiments demonstrate that macrophages from *Muc2^+/-^* females are more prone to anaerobic glycolysis, rather than oxidative phosphorylation, compared to C57BL/6 (Figure 3), which might provide a favorable metabolic state for M1 polarization under specific circumstances like pregnancy in combination with an external stimulus, like fucose (Figure 2) [67,68]. Apparently, fucose is one of the stimuli that can promote macrophage functions depending on their intrinsic properties. In agreement with this hypothesis, the two prototypical mouse strains with Th1- and Th2-type of the innate immune response demonstrated the opposite effect of fucose treatment on pregnancy outcome (Figure 4). This means that such an important process as macrophage polarization during pregnancy might be differentially affected by fucose depending on the immune status of these cells. Thus, macrophage polarization might at least partially explain the opposite effect of fucose on pregnancy in the mouse strains under study.

It was shown previously that inhibition of terminal fucosylation affects the differentiation of M1 macrophages and leads to the resolution of inflammation [69]. Fucose itself has been shown to inhibit macrophage M1 polarization and ameliorated DSS-induced acute colitis [70]. At the same time, in our previous study, fucose improved inflammation neither in acute, nor in chronic DSS-induced colitis [71], which might also be explained by the differences in the baseline microflora and immune system activation in mice at the two animal facilities. At the same time, to our knowledge, there is no data on the effects of fucose on macrophages in vitro. Our experiments demonstrated that macrophages can be directly affected by fucose as it induced phagocytosis in vitro (Figure 3A). Thus far, we do not know whether fucose acts via macrophage membrane receptors or directly interferes with metabolism. This question should further be addressed in order to understand the molecular mechanisms of fucose action on immune cells with a predisposition to Th-1 or Th-2 immune response. Altogether, these findings demonstrate that fucose causes a dramatic reduction in the reproductive performance in mice predisposed to Th2-type immune response or in mice lacking key intestinal commensal bacteria. Thus, fucose, a potentially beneficial molecule, is also a physiologically active compound that might have deleterious effects depending on the immune background and must be used with caution. 

## 5. Conclusions

This study shows that intestinal microbiota associated with chronic inflammation in ***Muc2^+/−^*** mice might affect pregnancy outcome when transferred to the otherwise healthy C57BL/6 animals. This might be explained, at least in part, by its effect on macrophage polarization and metabolism, as these cells actively participate in implantation and pregnancy progression. At the same time, fucose acts as a differential regulator of macrophage polarization depending on their intrinsic functional and metabolic programs. Thus, fucose exerts opposing effects on pregnancy development in mice with predisposition to Th-1 or Th-2 immune response.

## Data Availability

The data presented in this study are available on request from the corresponding author.
